# Endoscopic Gastric Band Removal

**DOI:** 10.3390/jcm12020617

**Published:** 2023-01-12

**Authors:** Thierry Manos, Anamaria Nedelcu, Patrick Noel, Viola Zulian, Marc Danan, Ramon Vilallonga, Sergio Carandina, Marius Nedelcu

**Affiliations:** 1 ELSAN—Clinique Bouchard, 13000 Marseille, France; 2 Centre Chirurgical de l’Obésité, ELSAN—Clinique Saint Michel, 83100 Toulon, France; 3 Mediclinic Airport Road Hospital, Abu Dhabi 48481, United Arab Emirates; 4 Universitat Autònoma de Barcelona, 08028 Barcelona, Spain; 5 Endocrine, Metabolic and Bariatric Unit, General Surgery Department, Hospital Vall d’Hebron, 08023 Barcelona, Spain

**Keywords:** laparoscopic adjustable gastric band, complication, migration, endoscopy

## Abstract

Background: Laparoscopic adjustable gastric band (LAGB) procedures have declined worldwide in recent years. A known complication is the intraluminal erosion of the prosthetic material. The endoscopic management of gastric band erosion represents the recommended approach nowadays, and it avoids any additional trauma to the gastric wall already damaged by the migration. The purpose of our study was to assess the feasibility of endoscopic management for intraluminal gastric band erosion following LAGB. Methods: From January 2009–December 2020, a total of 29 patients were retrospectively reviewed after undergoing endoscopic gastric band removal. The study included all consecutive patients who underwent endoscopic gastric band removal in this period. No patients were excluded from the study. Data on patient demographic characteristics, case history, operative details (procedural time, adverse events), and complications were reviewed retrospectively. Results: Twenty-nine patients underwent endoscopic gastric band removal: 22 women (75.8%) with a mean age of 45 years (range: 28–63) and mean Body Mass Index (BMI) of 31 ± 4.7 kg/m^2^ (range: 24–41). The average time to the identification of erosion after LAGB was 42 months (range: 28–137). The initial upper endoscopy found a migrated band of more than half of the diameter in 21 cases, less than a half but more than a third in seven cases and in one case, less than a third (use of a stent). Twenty-seven patients were successfully treated with endoscopic removal, and in two cases, the endoscopic approach failed, and laparoscopy was further performed. Conclusions: The endoscopic management of intraluminal erosion after LAGB can be safe and effective and should be considered the procedure of choice when treating this complication. The percentage of the band migration is important for the timing of the endoscopic removal.

## 1. Introduction

Currently, obesity is a chronic disease. It is important for the patient and their healthcare providers to understand this fact. The surgical treatment for this disease should be considered to be a staged approach. The use of a laparoscopic adjustable gastric band (LAGB) is a less radical procedure compared to the Roux-Y gastric bypass (RYGB) or laparoscopic sleeve gastrectomy (LSG), especially in the past, it has been chosen for the treatment of less extreme morbid obesity [1,2,3].

LAGB procedures have declined worldwide in recent years. In most of the national registers, the numbers of gastric band removals and revisions following LAGB have surpassed the implants [4]. A known complication was the intraluminal erosion of the prosthetic material. Initially, the perigastric technique was the most frequently used for adjustable gastric procedures [5]. In order to decrease the complication rate, the latter, the band was placed using the pars-flaccid technique [6]. It is often suggested that the retrogastric or the pars flaccid technique would have better results in terms of less slippage and less migration than the perigastric technique. It typically occurs many years after placement. The symptoms of erosion are variable but often include epigastric pain, dysphagia, reservoir infection, or no restriction despite aggressive band inflation.

The management of this complication can obviously be accomplished through surgery, which could be extremely challenging in these cases of migration, and the risk of postoperative complications could be extremely important. The advances in minimally invasive techniques have allowed clinicians to manage gastric band erosion endoscopically, providing improved patient outcomes [7]. The endoscopic management of gastric band erosion represents the recommended approach nowadays, and it avoids any additional trauma to the gastric wall already damaged by the migration. This represents an important aspect considering the high rate of revisional surgery following LAGB [8]. We report our experience as a regional referral center with the endoscopic management of intraluminal gastric band erosion following LAGB.

## 2. Materials and Methods

From January 2009 to July 2020, a total of 29 patients were retrospectively reviewed after undergoing endoscopic gastric band removal. All of the procedures were performed by a single endoscopist (T.M.) in two private hospitals. The study included all consecutive patients who underwent an endoscopic approach for gastric band removal in this period. No patients were excluded from the study. The data on the patient demographic characteristics, case history, operative details (procedural time and adverse events), and complications were reviewed retrospectively. We collected the following variables for reporting: original procedure, time to erosion, symptom presentation, details of the removal procedure, and postoperative complications. An informed consent document is typically used for all patients, and the study was approved by our institutional ethical committee.

**Figure 1:**
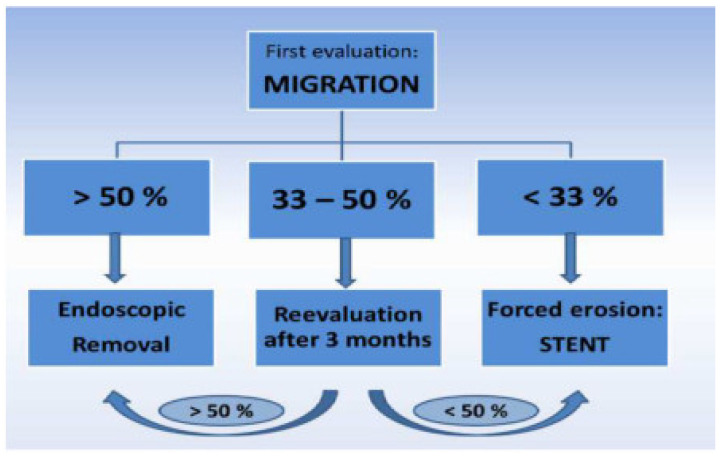
Algorithm for endoscopic gastric band removal.

Our decisional algorithm was synthetized, and it is shown in Figure 1. An upper endoscopy is always performed to evaluate the percentage of band migration. If the band had migrated more than half of its diameter, we proceeded directly with endoscopic band removal. If the band has migrated less than 50% but more than a third, a reevaluation upper endoscopy is performed in 3 months. Meanwhile, the band was gradually inflated depending on the patient’s symptoms. If the migration was less than one-third of the band’s diameter, a stent was deployed in order to induce more migration, and an upper endoscopy was performed after one month to reevaluate the migration rate.

### 2.1. Technique

**Figure 2:**
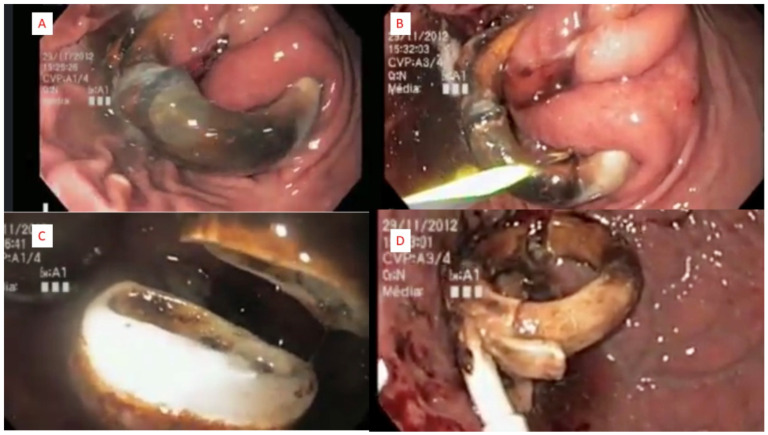
Operative technique: (**A**)—initial aspect; (**B**)—passage of the guidewire; (**C**)—complete sectioning; (**D**)—final aspect.

The connecting tube was sectioned, and the reservoir was removed before the procedure under local anesthesia or during the endoscopic procedure. In our technique, the single channel gastroscope (9.9 mm diameter) was used in all the cases. We first passed a 450 cm biliary guidewire (Boston Scientific Hydra Jagwire ST, Marlborough, MA, USA) around the eroded material. We pass the wire through a gastric band cutter (A.M.I. Gastric Band Cutter; CJ Medical, Haddenham, UK), and tightening the wire through this device cause the wire to sever the eroded band material. We then removed the band using either a snare or large endoscopic forceps. All of the operative steps are summarized in Figure 2. The removal of the band material was considered complete when there were no apparent missing portions of the band when examined ex vivo. No attempt was made to close the resulting mucosal defect. All of the procedures were performed under general anesthesia.

In France, the recommendation for bariatric revisional cases is to proceed with the 2-step approach. In the case of complication, such as migration, this approach represents no matter of discussions, and it is delayed to a minimum of 6 months. No patients underwent the removal of a partially eroded gastric band at the time of another bariatric procedure.

### 2.2. Statistical Analysis

The continuous demographic variables were expressed as mean ± standard deviation and range; categorical variables, as well as complications, were reported as numbers and percentages. The continuous outcome variables were generally reported as mean ± standard deviation and range. Descriptive statistics (simple counts and mean values) were used to report the complications and adverse effects.

## 3. Results

During the study period, 29 patients underwent endoscopic gastric band removal. There were 22 women (75.8%) and seven men (24.2%), with a median age of 45 years (range: 28–63). The overall mean body mass index (BMI) was 31 ± 4.7 kg/m^2^ (range: 24–41), and the mean preoperative weight was 81.3 ± 18.7 kg (range: 51–125 kg). Twenty-five patients (86.2%) had procedures performed on an outpatient basis. All of the patients have had an adjustable gastric band. Considering that in our region, there is an important number of patients with the band placed around the esophagus, it is worth mentioning that in all cases, the band was placed around the stomach. The average time to the identification of erosion after LAGB was 42 months (range 28–137 months). Regarding clinical symptomatology, we have identified the following:

- Weight regain for 17 patients (58.6%)
- Abdominal pain and fever with repeated port infection for nine patients (31%)
- Asymptomatic with an incidental finding on yearly upper GI swallow for three patients (10.3%)

The operative reports were also reviewed for the technique used for LAGB: perigastric—eight cases and pars flaccida—12 cases. In the rest of the nines cases, the operative report of the primary procedure was not available. The overall mean operative time for endoscopic removal was 31.2 ± 16.4 min (range: 7–83 min).

The initial upper endoscopy found a migrated band of more than half of the diameter in 21 cases, less than half but more than a third in seven cases, and in one case, less than a third. In this case, a 12-cm-long/20-mm-diameter stent (Taewoong Medical Co. Ltd., Goyang, Republic of Korea) was deployed for a period of one month. Consecutively the patient had a migration of more than half of the diameter, and the band was removed endoscopically.

Twenty-seven patients were successfully treated with endoscopic removal of the prosthetic materials using the gastric band cutter according to our algorithm (Figure 1). In two cases, the endoscopic approach failed, and laparoscopy was further performed. In one case, the band was sectioned by endoscopy, but the band buckle was entrapped in perigastric adherence. In the other case, the band was very rigid and impossible to be sectioned by the endoscopy. 

In another case, we encountered great difficulty in removing the band sectioned from the stomach at the level of the gastroesophageal junction. The decision taken was to deploy a stent in order to facilitate the removal. The stent was immediately removed after band extraction. Another option would be to continue to section the band. For the rest of the 26 patients, no additional adverse events were recorded during the endoscopy.

All of the patients were systematically followed by our multidisciplinary team at 1, 3, and 6 months after the procedure. A number of 18 patients out of 29 (62.1%) have had a revisional bariatric surgery of Roux en Y Gastric Bypass, except two patients who underwent sleeve gastrectomy.

## 4. Discussion

In order to evaluate the long-term results of LAGB, we should take into account only the patients with the band in place. The number of reoperations needed is very high, and the index of gastric band removal is increasing every year. Most reoperations are performed for reasons of weight regain despite intensive attempts of follow-up, but also due to the high rate of complications. The two most common complications are slippage and band erosion.

The etiology of band erosion is still debatable. The theories of gastric erosion range from unrecognized gastric injuries during band placement, gastric wall ischemia secondary to a tight band, port and band infection, excessive upper pouch pressure increase subsequent to improper food intake, and recurrent vomiting [9]. Although the true incidence of erosion is difficult to obtain, the reported rates for this long-term complication are between 1% and 10% [7,10,11,12,13,14,15]. The rates of migration depend on three factors:

1. The moment of the follow-up when these rates are reported is probably the most important factor, as long-term experiences are reporting higher percentages of gastric migration.
2. The type of the primary procedure banded RYGBP vs. LAGB.
3. The nature of the prosthetic material: polypropylene mesh vs. adjustable or nonadjustable band.

The percentage could be even higher, Himpens et al. [8] concluded that nearly one out of three patients experienced band erosion, and nearly 50% of the patients required the removal of their bands (contributing to a reoperation rate of 60%). In order to identify these long-term complications, the follow-up of the patients with LAGB must include yearly gastrografin swallow. The prevalence of migration increases over time because patients often remain asymptomatic for long periods or even will never become symptomatic. Considering the risk of band migration and according to certain authors, a routine gastroscopy should probably be discussed [16,17]. In our experience, the migration was suspected on upper GI swallow in all but two cases. In one case, the upper endoscopy was performed for severe gastroesophageal reflux symptoms. In the other case, the diagnosis was missed during the first follow-up visits and it was identified by another surgeon during the next visit in the same center. No systematic upper endoscopy is performed in the follow-up of LAGB cases in our department or in our region.

A new concept [18,19,20] of forced erosion involves placing a covered stent across a stenosis created by the partially migrated band. The erosion of the gastric band can be used for patient benefit in this situation. Over the weeks after stent placement, pressure necrosis of the gastric wall between the stent and band results in the forced erosion of the gastric band and allows the subsequent removal at the time of stent retrieval. In our algorithm (Figure 1), this approach is proposed when the migration is less than one-third or if the migration remains less than half after 3 months of reevaluation. In one patient, a stent was used to favorable induce more migration in order to facilitate the endoscopic removal.

The endoscopic approach was used successfully in 93.1% of cases, with two cases requiring surgery for band removal. This percentage is acceptable, with a recently published experience by Robinson et al. [21] of 64%, and it could be explained by two major factors: adequate preoperative evaluation with a good index of diagnosis and endoscopic expertise concentrated in high volume centers. Regarding the technical difficulty, we recorded a case when the endoscopic approach was not feasible. The connecting part of the band was entrapped in the gastric wall. The possibility of mucosal dissection was discussed, but the risk of increasing the gastric defect was considered too important. After further discussion with a surgical team, laparoscopic removal was decided. In the other case, the band was very rigid and impossible to be sectioned by the endoscopy. Spann et al. [22] have concluded that the management of LAGB erosion with a purely endoscopic method must not be considered if the band buckle was not visible. In this case, they would consider a hybrid method, combining the endoscopic and laparoscopic approaches. In our experience, a non-migrated band buckle should not be considered a contraindication to the purely endoscopic approach.

Two of the major limitations of our study, as for many other studies, are represented by the retrospective methodology of the study and the limited number of cases included. Our case experience of 29 patients could be considered limited, but the largest experience published was reported by Robinson et al. [21], with 22 cases of band migration and only 14 cases with complete endoscopic removal.

## 5. Conclusions

The endoscopic management of intraluminal erosion after LAGB can be safe and effective and should be considered the procedure of choice when treating this complication of adjustable gastric bands. The erosion of the band inside the gastric lumen allows for potential endoscopic removal without free intraabdominal perforation, compared with the surgical approach. It is important to have a certain expertise in advanced therapeutic endoscopy but also to respect an algorithm in order to better evaluate the results of the endoscopic approach. The percentage of band migration is important for the timing of endoscopic removal.

## Data Availability

Our data was included in SOFFCO registry that French society of bariatric surgery keeps confidential.
